# Interaction between influenza vaccine and statins affecting the risk of rhabdomyolysis in Taiwan: a nationwide case-centred analysis

**DOI:** 10.1016/j.eclinm.2025.103171

**Published:** 2025-04-10

**Authors:** Che-Yu Chen, Miyuki Hsing-Chun Hsieh, Wan-Ting Huang, Edward Chia-Cheng Lai

**Affiliations:** a School of Pharmacy, Institute of Clinical Pharmacy and Pharmaceutical Sciences, College of Medicine, National Cheng Kung University, Tainan 70101, Taiwan; b Population Health Data Centre, National Cheng Kung University, Tainan 70101, Taiwan; c Global Health Program, College of Public Health, National Taiwan University, Taipei 10055, Taiwan; d National Taiwan University Children’s Hospital, Taipei 10041, Taiwan

**Keywords:** Influenza vaccines, Statin, Rhabdomyolysis, Vaccine-drug interactions, Case-centred analysis, Taiwan

## Abstract

**Background:**

Literature suggests a potential interaction between influenza vaccination, statin use and rhabdomyolysis, but evidence is limited to case reports.

**Methods:**

Using out- and inpatient health records from Taiwan’s National Health Insurance Research Database (NHIRD) between January 2016 and December 2021, we retrospectively constructed a nationwide cohort of patients aged 50 years and older, first-ever diagnosed with rhabdomyolysis, focusing on those who received an influenza vaccine within the preceding one year. We applied a case-centred analysis to evaluate the interaction between statin use and influenza vaccination within specific risk intervals: 1–7 days and 8–14 days post-vaccination, as well as 30-day and 60-day windows for statin use prior to rhabdomyolysis diagnosis. The main outcome measures were odds ratios (ORs) for statin-associated rhabdomyolysis, stratified by timing of influenza vaccination.

**Findings:**

Among the 5,602 rhabdomyolysis cases analysed, 1,765 patients were exposed to statins within 30 days, and 1,838 patients were exposed within 60 days. 74 individuals were vaccinated within 7 days prior to their diagnosis, 30 of which were taking statins inside the 30-day interval, these individuals were found to be at a significantly higher risk of statin-related rhabdomyolysis (OR: 1.67, 95% confidence interval: 1.04–2.69). A similar risk was observed when the statin risk interval was extended to 60 days, 74 vaccinated rhabdomyolysis patients with 32 within the 60 day window (OR: 1.79, 95% confidence interval: 1.12–2.87). However, this increased risk was not observed among the 97 individuals (24 patients in the 30 day window and 26 in the 60 day) who received vaccination 8–14 days before rhabdomyolysis onset (OR: 0.85, 95% confidence interval: 0.53–1.36), and not in those vaccinated outside these risk intervals.

**Interpretation:**

Our results suggest a significant temporal association between recent influenza vaccination and increased risk of statin-associated rhabdomyolysis within 7 days post-vaccination. These findings highlight the need for healthcare providers to monitor for rhabdomyolysis symptoms following influenza vaccination in patients receiving statin therapy. Further confirmation in larger prospective international studies is warranted to better understand this potential association.

**Funding:**

National Science and Technology Council of Taiwan (NSTC 112-2628-B-006-003-; NSTC 113-2628-B-006-009-) and the 10.13039/501100004737National Health Research Institutes of Taiwan (NHRI-11A1-CG-CO-04-2225-1; NHRI-12A1-CG-CO-04-2225-1; NHRI-13A1-CG-CO-04-2225-1; NHRI-14A1-CG-CO-04-2225-1).

**Research in context**

**Evidence before this study**

Acute rhabdomyolysis following influenza vaccination in the elderly concurrently on statins has previously been reported; however, few studies have assessed potential interactions between the two exposures and their impact on the risk of rhabdomyolysis. We searched PubMed for studies up to 14 February 2025, using the following search terms: “rhabdomyolysis” AND “HMG-CoA reductase inhibitor OR statin” AND “(influenza AND vaccin∗) OR influenza vaccin∗”. Available evidence included only case reports and case series without large population-based studies investigating how influenza vaccination may interact with statin exposure to affect the risk of rhabdomyolysis.

**Added value of this study**

This nationwide study in Taiwan analysed 5,602 adults aged 50+ with rhabdomyolysis, including 1,765 on statins within 30 days and 1,838 within 60 days, as well as 74 vaccinated within 1–7 days and 97 within 8–14 days. This case-centred analysis of a rare event, based on a small sample, found an increased risk of statin-associated rhabdomyolysis for those vaccinated within 7 days, with adjusted odds ratios of 1.67 (95% CI: 1.04–2.69) for statin use within 30 days and 1.79 (95% CI: 1.12–2.87) for statin use within 60 days, while no increased risk was observed for vaccination 8–14 days prior. Though rare, these findings suggest a potential statin-vaccine interaction that warrants further investigation.

**Implications of all the available evidence**

We identified an interaction between recent influenza vaccination (1–7 days) and statins use, with an impact on rhabdomyolysis risk. Despite the potential increase in risk, rhabdomyolysis remains rare and often self-limiting; thus, this finding should not deter high-risk patients from receiving the influenza vaccine. Instead, these results highlight the need for healthcare providers to monitor for rhabdomyolysis symptoms in patients on statin therapy.

## Introduction

Rhabdomyolysis is characterized by the breakdown of skeletal muscle tissue, releasing muscle proteins and electrolytes into the bloodstream. This process of muscle necrosis can cause a wide range of clinical manifestations, such as myalgia, weakness and tea-coloured urine.^1^, ^2^, ^3^ Currently, a handful of case reports of acute rhabdomyolysis following influenza vaccines in older adult patients have raised concerns that the vaccines may occasionally cause rhabdomyolysis, 1–12 days post-vaccination.^4^, ^5^, ^6^, ^7^, ^8^ All of the involved patients concurrently used statins, which is a common non-traumatic cause of rhabdomyolysis, suggesting that the influenza vaccine might not be directly responsible for rhabdomyolysis. Instead, influenza vaccination may interact with statin exposure and thus affect the risk of rhabdomyolysis.

Taiwan’s initiated seasonal influenza mass vaccination campaigns in 1998.^9^ The annual programs target individuals at high risk of severe illness from influenza infection, such as those over age 65, for government-funded vaccination. Since 2016, this target group has been expanded to include those aged 50–64, with coverage by the vaccination program, in 2016, reaching 20.1% and 49.2% of persons aged 50–64 and 65+, respectively.^8^ Given that those aged 50+ are the predominant users of statin drugs due to their high prevalence of cardiovascular disease, the improved vaccination coverage for this priority group may increase the number of individuals exposed to both statins and influenza vaccines.^10^ Therefore, accurate assessment of the risk of rhabdomyolysis in this population is crucial.

Despite extensive previous research on side effects associated with statins,^11^, ^12^, ^13^, ^14^, ^15^ there is a paucity of studies investigating the real-world safety of statin treatment, particularly following influenza vaccination. Only the aforementioned case reports have revealed a possible relationship between statins, vaccination and rhabdomyolysis. However, their capacity to establish causality is limited as they are anecdotal, biased, and may not always be reliably confirmed. In this population-based study, we used Taiwan’s National Health Insurance Research Database (NHIRD) to investigate a potential effect modification on statins by influenza vaccination, and its impact on the risk of rhabdomyolysis.

## Methods

### Ethics

This study was conducted in accordance with the Declaration of Helsinki and the protocol was approved by the institutional review board of National Cheng Kung University Hospital (Project identification code B-ER-107-378).

### Study design

The exposures of interest included statin use and influenza vaccine uptake. Considering that both exposures of interest, and the outcome (i.e., rhabdomyolysis), exhibited varying degrees of seasonality (see Supplementary Information), we conducted the study using a case-centred design to address potential time-varying confounding.^16^^,^^17^ Case-centred analysis is a case-only design that compares the probability of exposure within a specified risk window among those who experienced events (or cases) to the entire population within the same sex and age strata, or other risk factor-matched strata. Case-centred analysis is similar to a case-control study without sampling, in which all available individuals, in most situations referring to the population with and without events, are utilized in the study. In this approach, each case is matched to all available subjects (or reference set) with the same age and sex on the event date of the case, from the entire population. The null hypothesis of this design is no association between the exposure and the outcome of interest; thus the probability of exposure within a specified time period is the same between cases and their reference sets. In our study, we hypothesized that the probability of statin exposure within the risk period was higher in the rhabdomyolysis cases, compared to their corresponding reference sets. This association may be modified if further stratified by prior vaccination status and timing.

### Study cohort

**Fig. 1:**
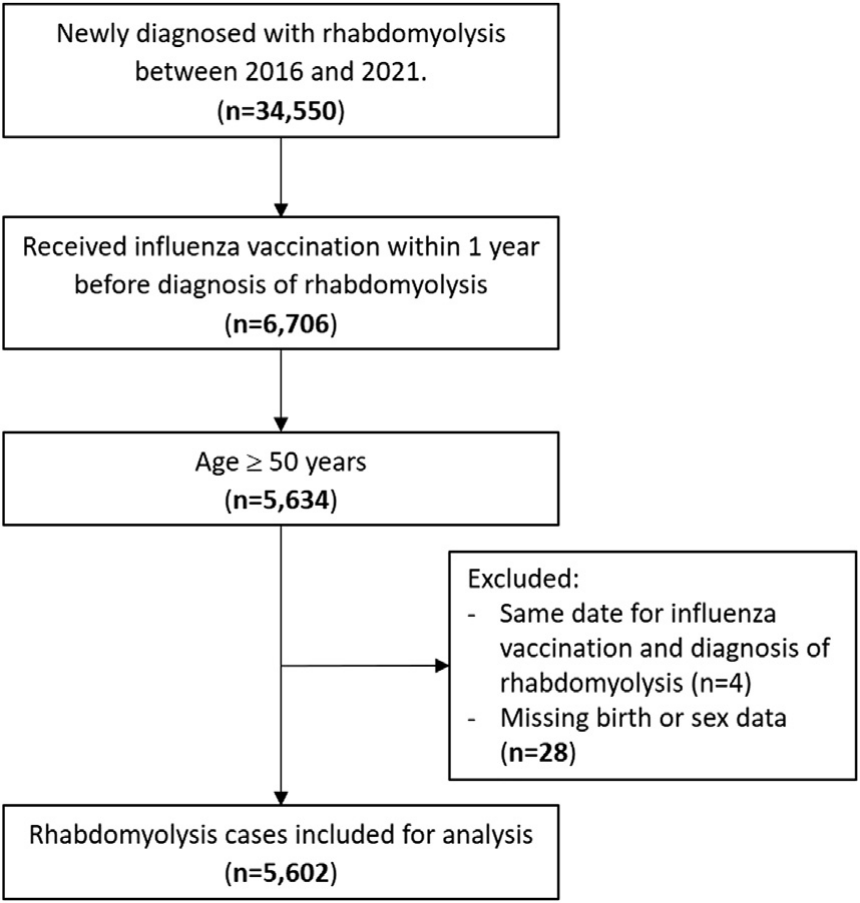
Study population selection.

We used data from Taiwan’s NHIRD from 2015 to 2021. Taiwan’s National Health Insurance program, implemented in 1995, is a universal, single-payer system providing mandatory coverage for all Taiwanese citizens. As of 2021, this health insurance program covered over 23 million people (more than 99% of the Taiwanese population).^18^ Claims data available in the NHIRD include diagnoses, prescriptions (including drugs and other medical interventions), examinations and procedures, gathered from hospitalizations, outpatient visits and emergency department visits. Providers who administer the government-purchased influenza vaccines to older adult patients (aged 50+) are reimbursed for procedures using a system involving drug numbers for individual vaccine brands. All individual data are analysed using an encrypted identification number to protect patient data privacy. Further detailed information about the NHIRD can be obtained elsewhere.^19^ Figure 1 presents the selection of the study cohort. The study enrolled case-patients above age 50 with first-ever diagnoses of rhabdomyolysis, identified using the *International Classification of Diseases, Tenth Revision, Clinical Modification* diagnosis code (ICD-10-CM M62.82), between 1 January 2016 and 31 December 2021. If patients were diagnosed with rhabdomyolysis more than once during the study period, we selected the earliest diagnosis in any setting as the event date. We considered cases as newly diagnosed if no identical rhabdomyolysis diagnosis preceded the event date within 365 days. We excluded patients younger than 50 years since the reimbursement for influenza vaccine did not fully apply to this age group, and their vaccination status could therefore not be reliably identified in the NHIRD. We defined the case index date as the event date, or date of rhabdomyolysis diagnosis, and for each case, we composed a reference set of subjects from the entire NHIRD population with the same sex and age ( ± 2 years) at the index date with the same calendar time as the case. Furthermore, to increase comparability between cases and reference sets, we included only subjects who had received influenza vaccine in the year preceding the index date, since studies have shown that health literacy and numerous other characteristics differ greatly between vaccinees and non-vaccinees.^20^

### Exposure measures

The exposures of interest were statins and influenza vaccine. All statin drugs available in Taiwan are reimbursed by the national health insurance program and we used Anatomical Therapeutic Chemical (ATC) codes to identify the statins used by each patient before the index date (Supplementary Table S1). Furthermore, we used ATC code J07BB02 and the domestic procedure code A2001C to identify patients’ influenza vaccination status. To assess rhabdomyolysis risk, we defined the risk interval of statin treatment as 1–30 days prior to the index date, based on published literature.^21^^,^^22^ Moreover, to assess rhabdomyolysis risk following vaccination, we examined two different risk intervals prior to the index date: 1–7 days and 8–14 days, depending on when individuals received the vaccine. Only patients with prescription dates within the defined risk periods were counted as exposed. We also excluded patients who received influenza vaccine on the date of rhabdomyolysis diagnosis since the temporal relationship between vaccination and outcome event could not be clearly established.

### Covariates and control of confounding

In addition to matching by age (±2 years) and sex, we further refined our control selection to better account for potential confounders and risk factors. Each included case was additionally matched to a subset of reference controls with identical rhabdomyolysis risk factors,^2^^,^^3^^,^^5^^,^^23^, ^24^, ^25^, ^26^, ^27^, ^28^, ^29^, ^30^, ^31^, ^32^, ^33^, ^34^, ^35^, ^36^, ^37^, ^38^, ^39^ including traumatic injury, heat exposure, hyperthermia, hypothyroidism, myopathy, epilepsy, stroke, poisoning, sepsis, water deprivation, electrolyte imbalances, and fibrate use within a predefined period before the event date. Detailed definitions are provided in Supplementary Table S2.

### Statistics

We reported continuous variables as means with standard deviations, and categorical variables as frequencies with percentages. After identifying rhabdomyolysis cases, we calculated the average annual incidence of rhabdomyolysis by dividing the mean number of cases per year by the total population of Taiwan, estimated at approximately 23 million during the study period. The case-centred approach applied a logistic regression model with offset term to calculate odds ratios (ORs) with 95% confidence intervals (CI) and p-values. This regression analysis examined whether there was a greater-than-expected number of patients among the rhabdomyolysis cases that had been exposed during the pre-specified risk interval(s).

**Fig. 2:**
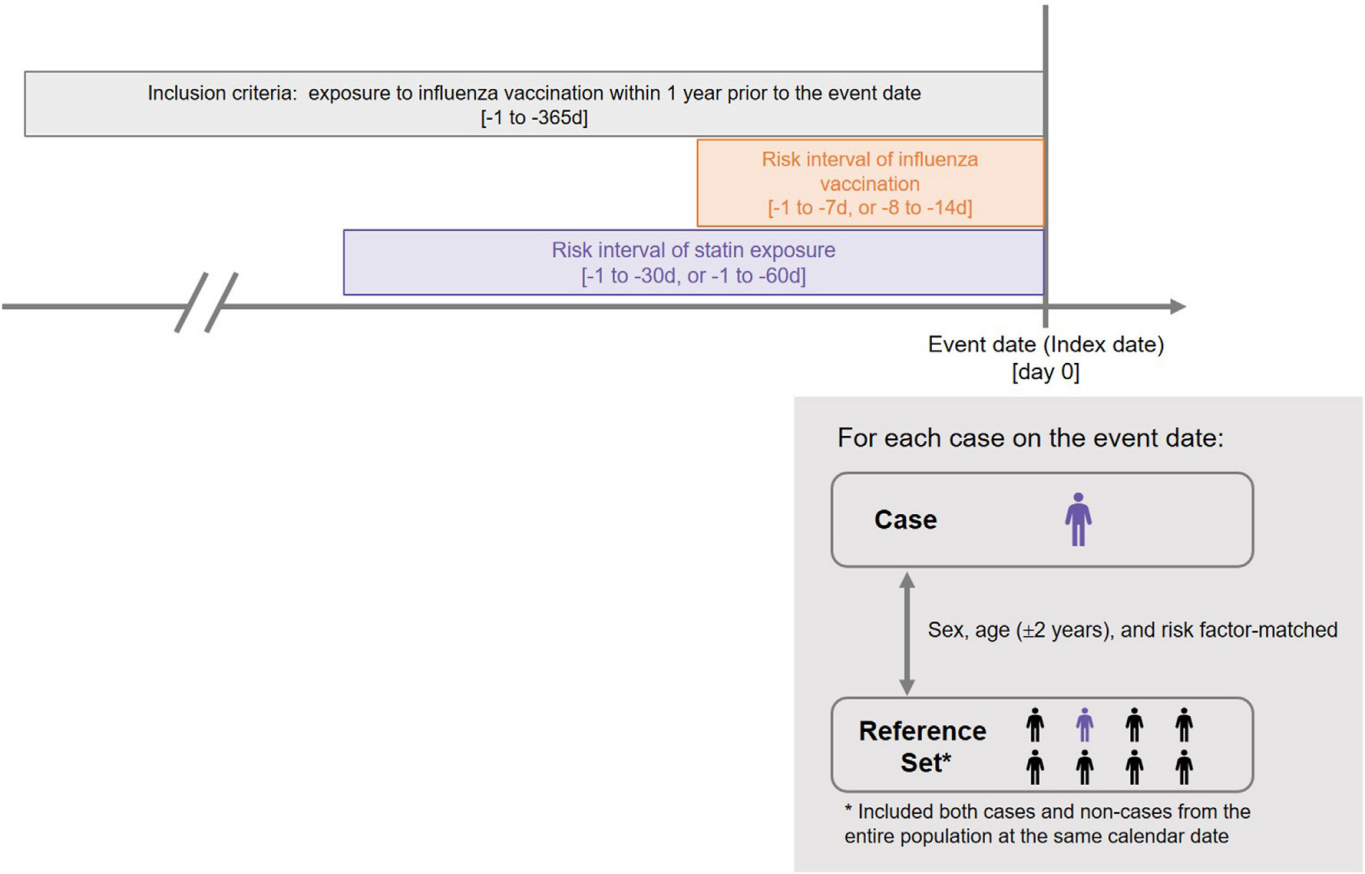
Case-centred design of this study.

The observed probability of exposure was either 1 or 0 for each case, given the statin exposure status during the pre-defined risk intervals prior to the index date (Fig. 2). A value of 1 indicated the presence of statin during the risk interval, whereas 0 indicated absence. To conduct such analysis, we first calculated the observed odds of cases being exposed to a statin medication inside the risk interval (1–30 days) prior to the index date. The expected odds were obtained from the corresponding matched reference set of each case and calculated as the odds of exposure within the risk interval. The logarithm of the expected odds of the exposure was then entered as the offset term in the logistic regression. Details of the application of the regression can be referenced elsewhere.^16^^,^^40^

To further explore potential interactions between influenza vaccination and statin use, we stratified the analysis into different time periods regarding vaccination status (with *vs.* without vaccination during the 1–7 days and 8–14 days preceding the index date) to assess changes in ORs. Additionally, to evaluate our findings’ robustness, we conducted a sensitivity analysis by extending the risk interval for statin exposure from 30 days to 60 days preceding the index date. To give consideration to potential changes in healthcare behaviours and practices during the pandemic years, we also performed a separate analysis restricted to the years preceding the COVID-19 pandemic (i.e., 2016–2019). All analyses were conducted using SAS version 9.4 with significance level set at 0.05. The study was conducted and reported in accordance with the STROBE guidelines.

### Declaration of AI-assisted technologies in the writing process

During the preparation of this work, the author(s) used ChatGPT to improve the fluency of the English and check for grammatical errors. After using this tool/service, the author(s) reviewed and revised the content as necessary and take(s) full responsibility for the final version of the publication.

### Role of the funding source

The funder of the study had no role in the design and conduct of the study; the collection, management, analysis and interpretation of the data; the preparation, review or approval of the manuscript; and the decision to submit the manuscript for publication.

## Results

We identified 34,550 patients newly diagnosed with rhabdomyolysis from January 1, 2016 to December 31, 2021, resulting in an average annual incidence of 0.25 cases per 1,000 population. Among these, 6,706 patients had received at least one influenza vaccine within 365 days prior to the index date. After excluding those younger than 50 years and others based on additional exclusion criteria, the study cohort consisted of 5,602 rhabdomyolysis cases, with a mean age of 71.5 years (standard deviation [SD]: 11.4), and 68% male (Fig. 1). For each case, the matched reference sets generated from the whole NHIRD population had a mean (±SD) number of 73,420 (±61,810) subjects. Among the cases, the most prevalent risk factors for rhabdomyolysis were stroke (13.2%), trauma (8.9%) and fibrate treatment (5.0%). All other risk factors accounted for less than 3% (Table 1).

**Table 1:** Characteristics of included rhabdomyolysis cases.

Characteristics	Cases No. (%)
N	5,602
Age, mean (SD), y	71.5 (11.4)
Age group, y	
50–64	1,744 (31.1)
65–79	2,389 (42.6)
≥80	1,469 (26.2)
Sex	
Male	3,781 (67.5)
Female	1,821 (32.5)
Risk factors for rhabdomyolysis	
Trauma	498 (8.9)
Heat exposure	12 (0.2)
Hyperthermia	0 (0)
Hypothyroidism	9 (0.2)
Myopathy	162 (2.9)
Epilepsy	61 (1.1)
Stroke	740 (13.2)
Poisoning	91 (1.6)
Sepsis	14 (0.2)
Water deprivation	16 (0.3)
Electrolyte imbalances	53 (0.9)
Fibrates	282 (5.0)
Distribution of statin and influenza vaccination exposure status	
Statin exposure within 1–30 day risk window^a^	1,765 (31.5)
Statin exposure within 1–60 day risk window^a^	1,838 (32.8)
Influenza vaccination with 1–7 day risk window^a^	74 (1.3)
Influenza vaccination with 8–14 day risk window^a^	97 (1.7)

a The risk window was defined as the days before the index date (i.e., the diagnosis date of rhabdomyolysis).

Table 2 presents the ORs for statin exposure during the pre-specified risk intervals for the cases, stratified by the different vaccination periods preceding the index date. Seventy-four cases received influenza vaccination during the 1–7 day period before the index date, generating an OR of 1.67 (95% CI: 1.04–2.69), after adjusting for the background exposure rate from the general population with similar risk profile. By contrast, 97 cases received influenza vaccination during the 8–14 day period before the index date, giving an OR of 0.85 (95% CI: 0.53–1.36). Patients not vaccinated during the 1–7 day or 8–14 day periods preceding rhabdomyolysis, however, showed no increase in ORs (OR: 1.02, 95% CI: 0.96–1.08 for days 1–7, and OR: 1.03, 95% CI: 0.97–1.09 for days 8–14) (Table 2).

**Table 2:** Rhabdomyolysis risk associated with statin exposure within different lengths of risk window, stratified by recent Influenza vaccination status, data from 2016 to 2021.

Influenza vaccine-exposed interval prior to the index date	Total number of cases	Number of cases inside the statin-exposed interval	OR (95% CI)	p-value
**Statin risk window: 1–30 days prior to the index date**				
Individuals who received influenza vaccine prior to the index date				
1–7 days prior	74	30	1.67 (1.04–2.69)	0.034
8–14 days prior	97	24	0.85 (0.53–1.36)	0.504
Individuals who did NOT receive influenza vaccine prior to the index date				
1–7 days prior	5,528	1,735	1.02 (0.96–1.08)	0.507
8–14 days prior	5,505	1,741	1.03 (0.97–1.09)	0.319
**Statin risk window: 1–60 days prior to the index date**				
Individuals who received influenza vaccine prior to the index date				
1–7 days prior	74	32	1.79 (1.12–2.87)	0.015
8–14 days prior	97	26	0.91 (0.58–1.44)	0.698
Individuals who did NOT receive influenza vaccine prior to the index date				
1–7 days prior	5,528	1,806	1.03 (0.98–1.09)	0.260
8–14 days prior	5,505	1,812	1.04 (0.99–1.11)	0.144

The sensitivity analysis using a statin-exposed risk interval of 60 days produced similar results (Table 2). The rhabdomyolysis risk remained higher (OR: 1.79, 95% CI: 1.12–2.87) among patients who received influenza vaccination within 1–7 days before the index date. The increase in OR, however, was not observed for those receiving the vaccine in the preceding 8–14 days (OR: 0.91, 95% CI: 0.58–1.44), and also not among those not receiving the vaccine (OR: 1.03, 95% CI: 0.98–1.09 for days 1–7, and OR: 1.04, 95% CI: 0.99–1.11 for days 8–14) (Table 2).

The analysis restricted to the pre-pandemic years 2016–2019 yielded consistent results (Table 3). The rhabdomyolysis risk was higher (OR: 2.00, 95% CI: 1.05–3.82) among patients who received an influenza vaccine within 1–7 days before the index date. However, this increase in OR was not observed in those who received the vaccine 8–14 days prior (OR: 1.02, 95% CI: 0.55–1.88), nor among those who did not receive the vaccine (OR: 0.98, 95% CI: 0.91–1.06 for days 1–7, and OR: 0.99, 95% CI: 0.92–1.07 for days 8–14) within the pre-specified time periods (Table 3). The analysis of the pandemic years 2020–2021 is presented in Supplementary Table S4.

**Table 3:** Rhabdomyolysis risk associated with statin exposure within different lengths of risk window, stratified by recent Influenza vaccination status, data from 2016 to 2019.

Influenza vaccine-exposed interval prior to the index date	Total number of cases	Number of cases inside the statin-exposed interval	OR (95% CI)	p-value
**Statin risk window: 1–30 days prior to the index date**				
Individuals who received influenza vaccine prior to the index date				
1–7 days prior	39	16	2.00 (1.05–3.82)	0.036
8–14 days prior	56	14	1.02 (0.55–1.88)	0.957
Individuals who did NOT receive influenza vaccine prior to the index date				
1–7 days prior	3,325	947	0.98 (0.91–1.06)	0.685
8–14 days prior	3,308	949	0.99 (0.92–1.07)	0.853
**Statin risk window: 1–60 days prior to the index date**				
Individuals who received influenza vaccine prior to the index date				
1–7 days prior	39	18	2.35 (1.24–4.46)	0.009
8–14 days prior	56	15	1.07 (0.59–1.96)	0.815
Individuals who did NOT receive influenza vaccine prior to the index date				
1–7 days prior	3,325	997	1.01 (0.94–1.09)	0.801
8–14 days prior	3,308	1,000	1.02 (0.95–1.10)	0.610

## Discussion

Our study was the first population-based study to investigate a potential effect modification by influenza vaccination of the statin-related risk of rhabdomyolysis. Our study indicated a significant temporal association, whereby patients vaccinated against influenza within the preceding 7 days exhibited an increased risk of statin-associated rhabdomyolysis. This increased risk, however, was diminished both in those who received influenza vaccine in the 8–14 days preceding the event, and in those vaccinated outside these risk windows. These findings suggested a potential interaction between recent influenza vaccination and the use of statins in relation to rhabdomyolysis risk. Given the rarity of rhabdomyolysis and the observational nature of our study, caution is needed when interpreting these results. The sensitivity analysis showed consistent results when the risk interval for statin exposure was changed from 30 to 60 days, supporting the robustness of our analysis. However, further research is needed to validate these findings and explore the underlying mechanisms.

Our main result suggested that recent exposure to influenza vaccination may increase the risk of statin-associated rhabdomyolysis. While influenza infections are commonly reported as viral causes of myositis or even rhabdomyolysis, muscle-related adverse events associated with influenza vaccination are rarely reported.^41^^,^^42^ Previous literature includes clinical reports describing cases of rhabdomyolysis with renal complications believed to be associated with influenza vaccination. These cases share a common risk factor: concurrent statin therapy. It is hypothesized that influenza vaccination may act as a trigger for rhabdomyolysis in patients on statin therapy. Although the incidence of influenza vaccine-exacerbated rhabdomyolysis is expected to be very rare, clinicians must remain aware of potential risks, given the potential development of kidney failure requiring renal replacement therapy or transplantation. Patients receiving influenza vaccination should be educated to promptly report any muscle-related symptoms such as myalgia or muscle weakness, especially those on statins or with other risk factors, to enable early detection and management.

We observed that the risk of rhabdomyolysis appeared higher in patients receiving vaccination within the 7 days preceding a rhabdomyolysis event. This finding regarding time-to-onset is consistent with a study utilizing the WHO-Vigibase and Vaccine Adverse Event Reporting System (VAERS) database to collect adverse event reports following immunization.^43^ The study found an average time-to-onset of rhabdomyolysis and myopathy of 8.4 days in 209 cases. Similarly, according to several aforementioned case reports,^4^, ^5^, ^6^, ^7^ the time-to-onset of rhabdomyolysis or related muscle symptoms following influenza vaccination ranged from 1 to 12 days.

The sensitivity analysis restricted to the years 2016–2019 yielded a higher OR for statin exposed cases within defined risk intervals, compared to their reference sets. This finding reinforced the results of the main analysis using data from 2016 to 2021, and supported our hypothesis that influenza vaccination might increase the risk of statin-associated rhabdomyolysis through a potential vaccine-drug interaction. The significant risk diminished in the years 2020–2021 (Supplementary Table S4), possibly due to the COVID-19 pandemic influencing healthcare-related behaviours and disease patterns.

The mechanism underlying the interaction between vaccines and medications remains unclear, and relevant discussion is limited. Some studies have suggested that the production of interferon-γ (INF-γ) after vaccination may affect metabolism of certain medications.^44^^,^^45^ In a previous case report, carbamazepine toxicity was found after influenza vaccination, suggesting that INF-γ may interfere with hepatic CYP3A4 enzymes, which are mainly responsible for metabolism of carbamazepine as well as multiple statins.^46^ Studies have shown that different CYP3A4 polymorphisms are associated with varying risks of increased statin plasma levels or treatment outcomes, suggesting genetic variations in CYP3A4 may yield enzymatic variants that contribute to inter-individual differences in drug metabolism.^47^, ^48^, ^49^ By contrast, other studies have found that INF-γ production has only minor or no effect on CYP2C9.^50^, ^51^, ^52^ Further research should investigate whether interference in medication metabolism plays a significant role in potential interactions between influenza vaccination and statins.

The study found that recent influenza vaccination may be associated with increased odds of statin-associated rhabdomyolysis. However, the absolute risk remains low due to the rarity of the event, and the findings should not cause undue concern. Rhabdomyolysis is a severe condition that can lead to kidney failure or death if not promptly managed. Our research primarily identified rhabdomyolysis cases via the ICD code, suggesting that these were likely severe instances. Patients with subclinical rhabdomyolysis or those with myopathy not progressing to rhabdomyolysis were not captured, hinting that actual incidence may be higher. Effective early treatment involving aggressive intravenous fluids typically results in full renal recovery. Further research is needed to confirm the study findings and better understand the potential relationship between influenza vaccination and statin-associated rhabdomyolysis. Therefore, increasing awareness among clinicians and patients regarding the risks and early symptoms of rhabdomyolysis is essential.

In real-world practice, healthcare professionals routinely review patients’ conditions before vaccination, being especially vigilant for immune system-related diseases, to avoid adverse events. However, drug-vaccine interactions may often be overlooked in clinical settings. Our findings draw attention to a potential vaccine-drug interaction, emphasizing the need for future real-world prospective analysis. Since the benefits of vaccination outweigh the relatively limited increased risk of statin-associated rhabdomyolysis, it is advisable for statin users to accept close monitoring for symptoms of myopathy, rather than forgoing influenza vaccination. The findings also suggest a potential need for pharmacist consultation when developing public vaccination plans, and electronic systems to automatically screen for a broad range of current drug uses and possible vaccine interactions should be considered in the future.

The strength of this study was its utilization of a nationwide database. The NHIRD provides a high level of representativeness, as it includes all individuals aged 50 and above who are eligible for government-funded annual seasonal influenza vaccination. This comprehensive inclusion permitted a thorough evaluation of the data. However, the findings may not directly apply to younger populations or countries with different healthcare systems or influenza vaccination strategies.

The study also had some limitations. First, seasonality in exposures and outcomes can lead to time-varying confounding bias in observational studies. In our research, the exposures were statin use with influenza vaccine as an effect modifier. We hypothesized that statin use might follow seasonal trends, since it is common in patients with cardiovascular events, which are more prevalent in winter. Indeed, our data revealed a distinct seasonal pattern in statin utilization (see Supplementary Information). Similarly, influenza vaccination exhibits a natural seasonal pattern,^53^ typically beginning and peaking in October, following natural epidemic trends and aligned with government-funded vaccination programs. Additionally, in Taiwan, with an annual incidence of approximately 0.25 per thousand people, rhabdomyolysis shows a seasonal peak during the summer (see Statistics in Methods section and Supplementary Information). To accommodate this varying seasonality in both exposures and outcomes, we adopted a case-centred analysis, effectively addressing potential confounding, controlling for time-varying factors and adjusting for the seasonality. Moreover, the case-centred analysis served as a case-only design, allowing for inclusion of all cases in the analysis, preserving high statistical power and minimizing random errors, particularly in the study of low-incidence outcomes such as rhabdomyolysis. Second, the ICD-10-CM codes for rhabdomyolysis in the NHIRD have not been validated.^54^ However, given that rhabdomyolysis represents a specific and severe form of muscle damage it was unlikely to have been overestimated. Hence, the outcome’s positive predictive value can be expected to be high. Furthermore, we acknowledge that misclassification of the outcome could have biased the results towards null. However, the fact that our findings revealed a significant risk, despite these concerns, indicates that this potential limitation is unlikely to have affected the overall conclusion of the study. Finally, our matching and adjustment may not have accounted for all potential covariates, such as alcohol consumption and exercise, which could introduce residual confounding. Additionally, we did not include medications beyond statins and fibrates that could increase the risk of rhabdomyolysis (e.g., psychiatric agents or antihistamines) in this study. Although these medications have been reported to be associated with rhabdomyolysis, they are typically implicated in cases of overdose or intoxication,^3^ which we have already considered (i.e., poisoning) in our analysis.

In summary, our study provides insights into a potential interaction between influenza vaccination and statin use, suggesting a short-term increased likelihood of rhabdomyolysis in older adults following vaccination. Clinicians may consider informing patients on statin therapy to be mindful of any muscle-related symptoms after vaccination, though the overall risk remains low. Further research is needed to confirm these findings, better understand the underlying mechanisms, and assess whether any adjustments in vaccination timing could be beneficial for statin users.

## Contributors

ECCL, WTH and CYC conceptualized and developed the research question. CYC and MHCH designed the study protocol. CYC drafted the manuscript. CYC, MHCH and ECCL are the guarantors. CYC, MHCH, WTH and ECCL edited the study protocol, interpreted the results, and reviewed the manuscript. MHCH wrote the statistical analysis plan, conducted the statistical analysis, and revised the manuscript. All authors proofread the final version of the manuscript and consented to its submission. The corresponding author attests that all listed authors meet authorship criteria and that no others meeting the criteria have been omitted. CYC and MHCH have accessed and verified the data, and all authors were responsible for the decision to submit the manuscript.

## Data sharing statement

The authors applied for and accessed the data from the data centre of the Ministry of Health and Welfare in Taiwan. Researchers who are citizen of Taiwan and are interested in accessing this dataset could submit a formal application to the Taiwan Ministry of Health and Welfare to request access (https://dep.mohw.gov.tw/DOS/cp-2516-59203-113.html). Data dictionary can be accessed here: https://dep.mohw.gov.tw/DOS/lp-2503-113-xCat-DOS_dc002.html.

## Declaration of interests

This study was supported in part by grants from the National Science and Technology Council of Taiwan (NSTC 112-2628-B-006-003-; NSTC 113-2628-B-006-009-) and the National Health Research Institutes of Taiwan (NHRI-11A1-CG-CO-04-2225-1; NHRI-12A1-CG-CO-04-2225-1; NHRI-13A1-CG-CO-04-2225-1; NHRI-14A1-CG-CO-04-2225-1) awarded to ECCL, but the funder had no role in the design and conduct of the study The other authors declare no competing interests.

## References

[1] Warren J.D., Blumbergs P.C., Thompson P.D. (2002). Rhabdomyolysis: a review. Muscle Nerve.

[2] Huerta-Alardín A.L., Varon J., Marik P.E. (2004). Bench-to-bedside review: rhabdomyolysis – an overview for clinicians. Crit Care.

[3] Torres P.A., Helmstetter J.A., Kaye A.M., Kaye A.D. (2015). Rhabdomyolysis: pathogenesis, diagnosis, and treatment. Ochsner J.

[4] Callado R.B., Ponte Carneiro T.G., da Cunha Parahyba C.C., de Alcantara Lima N., da Silva Junior G.B., de Francesco Daher E. (2013). Rhabdomyolysis secondary to influenza A H1N1 vaccine resulting in acute kidney injury. Trav Med Infect Dis.

[5] Plotkin E., Bernheim J., Ben-Chetrit S., Mor A., Korzets Z. (2000). Influenza vaccine--a possible trigger of rhabdomyolysis induced acute renal failure due to the combined use of cerivastatin and bezafibrate. Nephrol Dial Transplant.

[6] Raman K.S., Chandrasekar T., Reeve R.S., Roberts M.E., Kalra P.A. (2006). Influenza vaccine-induced rhabdomyolysis leading to acute renal transplant dysfunction. Nephrol Dial Transplant.

[7] Shah S.V., Reddy K. (2010). Rhabdomyolysis with acute renal failure triggered by the seasonal flu vaccination in a patient taking simvastatin. BMJ Case Rep.

[8] Novati R., Nebiolo P.E., Galotto C., Mastaglia M., Manes M. (2014). Acute renal failure after influenza vaccination: a case report. J Prev Med Hyg.

[9] Meyer D., Shearer M.P., Chih Y.C., Hsu Y.C., Lin Y.C., Nuzzo J.B. (2018). Taiwan’s annual seasonal influenza mass vaccination program-lessons for pandemic planning. Am J Public Health.

[10] Hsieh H.C., Hsu J.C., Lu C.Y. (2017). 10-year trends in statin utilization in Taiwan: a retrospective study using Taiwan’s National Health Insurance Research Database. BMJ Open.

[11] Thompson P.D., Clarkson P., Karas R.H. (2003). Statin-associated myopathy. JAMA.

[12] Graham D.J., Staffa J.A., Shatin D. (2004). Incidence of hospitalized rhabdomyolysis in patients treated with lipid-lowering drugs. JAMA.

[13] Mansi I., Frei C.R., Pugh M.J., Makris U., Mortensen E.M. (2013). Statins and musculoskeletal conditions, arthropathies, and injuries. JAMA Intern Med.

[14] Rosenson R.S., Baker S.K., Jacobson T.A., Kopecky S.L., Parker B.A. (2014). The national lipid association’s muscle safety expert P. An assessment by the statin muscle safety task force: 2014 update. J Clin Lipidol.

[15] Stroes E.S., Thompson P.D., Corsini A. (2015). Statin-associated muscle symptoms: impact on statin therapy—European atherosclerosis society consensus panel statement on assessment, aetiology and management. Eur Heart J.

[16] Fireman B., Lee J., Lewis N., Bembom O., van der Laan M., Baxter R. (2009). Influenza vaccination and mortality: differentiating vaccine effects from bias. Am J Epidemiol.

[17] Baker M.A., Lieu T.A., Li L. (2015). A vaccine study design selection framework for the postlicensure rapid immunization safety monitoring program. Am J Epidemiol.

[18] National health insurance annual statistical report 2019. https://www.mohw.gov.tw/cp-4995-56604-2.html.

[19] Hsieh C.Y., Su C.C., Shao S.C. (2019). Taiwan’s national health insurance research database: past and future. Clin Epidemiol.

[20] Biasio L.R. (2017). Vaccine hesitancy and health literacy. Hum Vaccin Immunother.

[21] Mendes P., Robles P.G., Mathur S. (2014). Statin-induced rhabdomyolysis: a comprehensive review of case reports. Physiother Can.

[22] Akimoto H., Negishi A., Oshima S. (2018). Onset timing of statin-induced musculoskeletal adverse events and concomitant drug-associated shift in onset timing of MAEs. Pharmacol Res Perspect.

[23] Nelson D.A., Deuster P.A., Carter R., Hill O.T., Wolcott V.L., Kurina L.M. (2016). Sickle cell trait, rhabdomyolysis, and mortality among U.S. Army soldiers. N Engl J Med.

[24] (2021). Update: exertional rhabdomyolysis, active component, U.S. Armed Forces, 2016-2020. MSMR.

[25] Cervellin G., Comelli I., Lippi G. (2010). Rhabdomyolysis: historical background, clinical, diagnostic and therapeutic features. Clin Chem Lab Med.

[26] Kraeva N., Sapa A., Dowling J.J., Riazi S. (2017). Malignant hyperthermia susceptibility in patients with exertional rhabdomyolysis: a retrospective cohort study and updated systematic review. Can J Anaesth.

[27] Reddy J.I., Cooke P.J., van Schalkwyk J.M., Hannam J.A., Fitzharris P., Mitchell S.J. (2015). Anaphylaxis is more common with rocuronium and succinylcholine than with atracurium. Anesthesiology.

[28] Salehi N., Agoston E., Munir I., Thompson G.J. (2017). Rhabdomyolysis in a patient with severe hypothyroidism. Am J Case Rep.

[29] Lin C.L., Wu S.Y., Huang W.T. (2019). Subsequent thyroid disorders associated with treatment strategy in head and neck cancer patients: a nationwide cohort study. BMC Cancer.

[30] van Gerwen M., Alsen M., Little C. (2020). Outcomes of patients with hypothyroidism and COVID-19: a retrospective cohort study. Front Endocrinol.

[31] Thvilum M., Brandt F., Brix T.H., Hegedüs L. (2018). No evidence of a causal relationship between hypothyroidism and glaucoma: a Danish nationwide register-based cohort study. PLoS One.

[32] Paternostro C., Gopp L., Tomschik M. (2021). Incidence and clinical spectrum of rhabdomyolysis in general neurology: a retrospective cohort study. Neuromuscul Disord.

[33] Melli G., Chaudhry V., Cornblath D.R. (2005). Rhabdomyolysis: an evaluation of 475 hospitalized patients. Medicine (Baltim).

[34] Mishra A., Dave N. (2013). Acute renal failure due to rhabdomyolysis following a seizure. J Family Med Prim Care.

[35] Talaie H., Emam-Hadi M., Panahandeh R., Hassanian-Moghaddam H., Abdollahi M. (2008). On the mechanisms underlying poisoning-induced rhabdomyolysis and acute renal failure. Toxicol Mech Methods.

[36] Kumar A.A., Bhaskar E., Palamaner Subash Shantha G., Swaminathan P., Abraham G. (2009). Rhabdomyolysis in community acquired bacterial sepsis--a retrospective cohort study. PLoS One.

[37] Lane R., Phillips M. (2003). Rhabdomyolysis. BMJ.

[38] Amanzadeh J., Reilly R.F. (2006). Hypophosphatemia: an evidence-based approach to its clinical consequences and management. Nat Clin Pract Nephrol.

[39] Wu J., Song Y., Li H., Chen J. (2009). Rhabdomyolysis associated with fibrate therapy: review of 76 published cases and a new case report. Eur J Clin Pharmacol.

[40] Rowhani-Rahbar A., Klein N.P., Lewis N. (2012). Immunization and Bell’s palsy in children: a case-centered analysis. Am J Epidemiol.

[41] Gibson S.B., Majersik J.J., Smith A.G., Bromberg M.B. (2013). Three cases of acute myositis in adults following influenza-like illness during the H1N1 pandemic. J Neurosci Rural Pract.

[42] Naderi A.S.A., Palmer B.F. (2006). Rhabdomyolysis and acute renal failure associated with influenza virus type B infection. Am J Med Sci.

[43] Carnovale C., Raschi E., Leonardi L. (2018). No signal of interactions between influenza vaccines and drugs used for chronic diseases: a case-by-case analysis of the vaccine adverse event reporting system and vigibase. Expert Rev Vacc.

[44] Aitken A.E., Morgan E.T. (2007). Gene-specific effects of inflammatory cytokines on cytochrome P450 2C, 2B6 and 3A4 mRNA levels in human hepatocytes. Drug Metab Dispos.

[45] Pellegrino P., Clementi E., Capuano A., Radice S. (2015). Can vaccines interact with drug metabolism?. Pharmacol Res.

[46] Robertson W.C. (2002). Carbamazepine toxicity after influenza vaccination. Pediatr Neurol.

[47] Becker M.L., Visser L.E., van Schaik R.H., Hofman A., Uitterlinden A.G., Stricker B.H. (2010). Influence of genetic variation in CYP3A4 and ABCB1 on dose decrease or switching during simvastatin and atorvastatin therapy. Pharmacoepidemiol Drug Saf.

[48] Jamil K., Kandula V., Kandula R., Asimuddin M., Joshi S., Yerra S.K. (2014). Polymorphism of CYP3A4∗2 and eNOS genes in the diabetic patients with hyperlipidemia undergoing statin treatment. Mol Biol Rep.

[49] Maslub M.G., Radwan M.A., Daud N.A.A., Sha’aban A. (2023). Association between CYP3A4/CYP3A5 genetic polymorphisms and treatment outcomes of atorvastatin worldwide: is there enough research on the Egyptian population?. Eur J Med Res.

[50] MacCallum P., Madhani M., Mt-Isa S., Ashby D. (2007). Lack of effect of influenza immunisation on anticoagulant control in patients on long-term warfarin. Pharmacoepidemiol Drug Saf.

[51] Jackson M.L., Nelson J.C., Chen R.T., Davis R.L., Jackson L.A. (2007). Vaccines and changes in coagulation parameters in adults on chronic warfarin therapy: a cohort study. Pharmacoepidemiol Drug Saf.

[52] Soontornpun A., Manoyana N., Apaijai N. (2020). Influenza immunization does not predominantly alter levels of phenytoin, and cytochrome P-450 enzymes in epileptic patients receiving phenytoin monotherapy. Epilepsy Res.

[53] Hsieh Y.C., Chen H.Y., Yen J.J. (2005). Influenza in Taiwan: seasonality and vaccine strain match. J Microbiol Immunol Infect.

[54] Huang Y.-T., Wei T., Huang Y.-L., Wu Y.-P., Chan K.A. (2023). Validation of diagnosis codes in healthcare databases in Taiwan, a literature review. Pharmacoepidemiol Drug Saf.

