# Supplementary material for: Interaction between influenza vaccine and statins affecting the risk of rhabdomyolysis in Taiwan: a nationwide case-centred analysis
Source: eClinicalMedicine. 2025 Apr 10;82:103171. doi: 10.1016/j.eclinm.2025.103171 (PMC12018050; doi:10.1016/j.eclinm.2025.103171)
Supplement: Supplementary Information and Tables S1–S4 [file mmc1.docx]

**Supplementary Materials**

| **Item** | **Title** | **Page number** |
| --- | --- | --- |
| Supplementary information | [Seasonality of statins exposure, influenza vaccinations and rhabdomyolysis events](#Supp_info) | Page 2 |
| Table S1 | [Included statins](#TS1) | Page 5 |
| Table S2 | [Risk factors for rhabdomyolysis](#TS2) | Page 6 |
| Table S3 | [Summary of case reports from literature regarding rhabdomyolysis following influenza vaccination](#TS3) | Page 7 |
| Table S4 | [Rhabdomyolysis risk associated with statin exposure within different risk windows, stratified by recent Influenza vaccination status; data from 2020-2021](#TS4) | Page 8 |

**Supplementary information: Seasonality of statins exposure, influenza vaccinations and rhabdomyolysis events**

**Seasonality of exposure to statins**

| 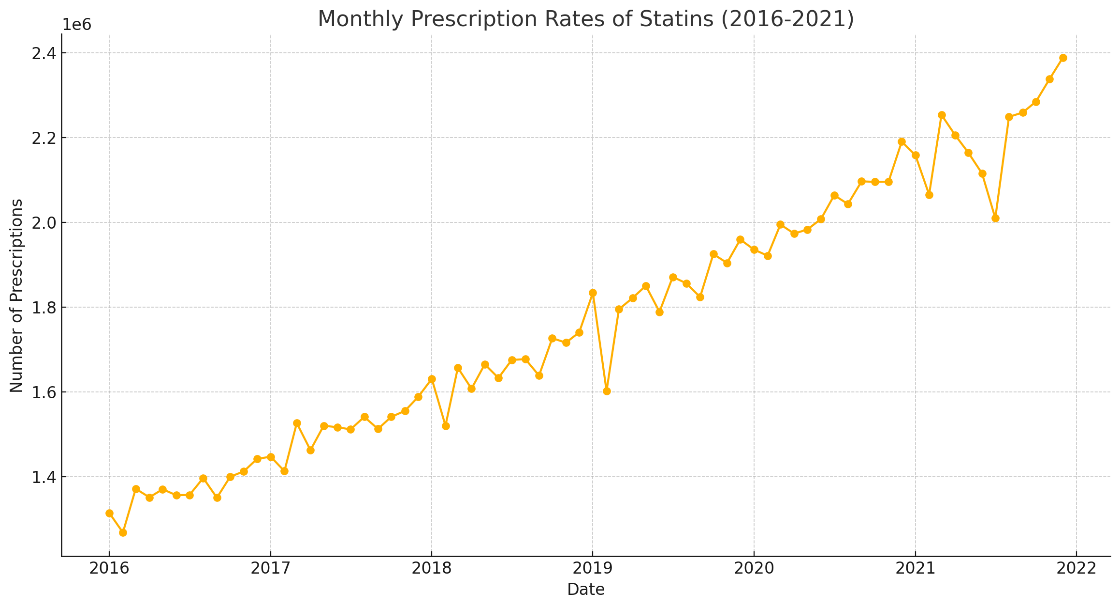 | 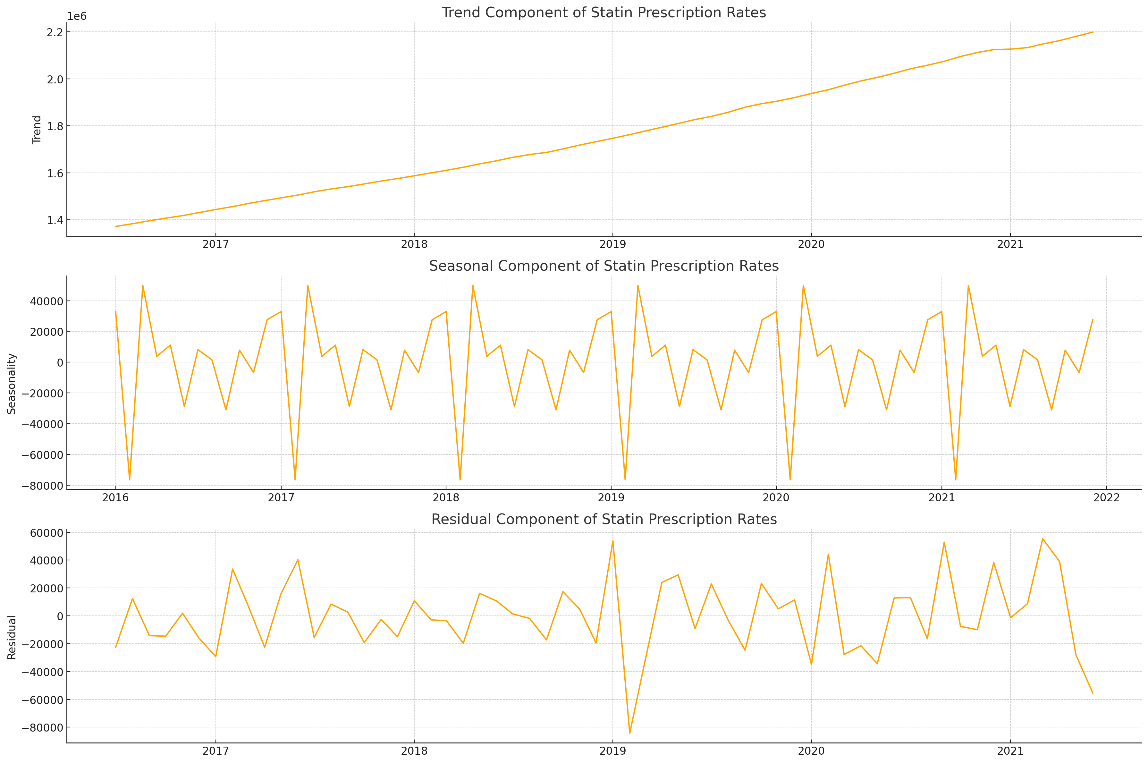 |
| --- | --- |
| Monthly Statin Prescriptions from 2016 to 2021 (**Observed**) | Decomposition of the statin prescriptions data (**Decomposed**) |

The seasonal decomposition of the statin prescriptions data provides four key components:

1. **Observed**: The original data, showing the total number of statin prescriptions per month.

**Decomposed**:

2. **Trend**: This indicates the long-term progression of the series, abstracting from the seasonal and random components. It reveals an overall increasing trend in statin prescriptions over the years.

3. **Seasonal**: This component captures the regular pattern of variability within a year. The plot shows a clear seasonal pattern, with certain peaks and troughs repeating annually.

4. **Residual**: These are the irregular components or 'noise' that remain after the trend and seasonal components are removed. The residuals do not show any specific pattern, suggesting that the trend and seasonal components explain most of the variability in the data.

The presence of a distinct and repeating pattern in the seasonal component suggests that statin prescription does exhibit seasonality. This means that the number of statin prescriptions varies in a somewhat predictable pattern throughout the year, which is repeated annually.

**Seasonality of influenza vaccination**

| 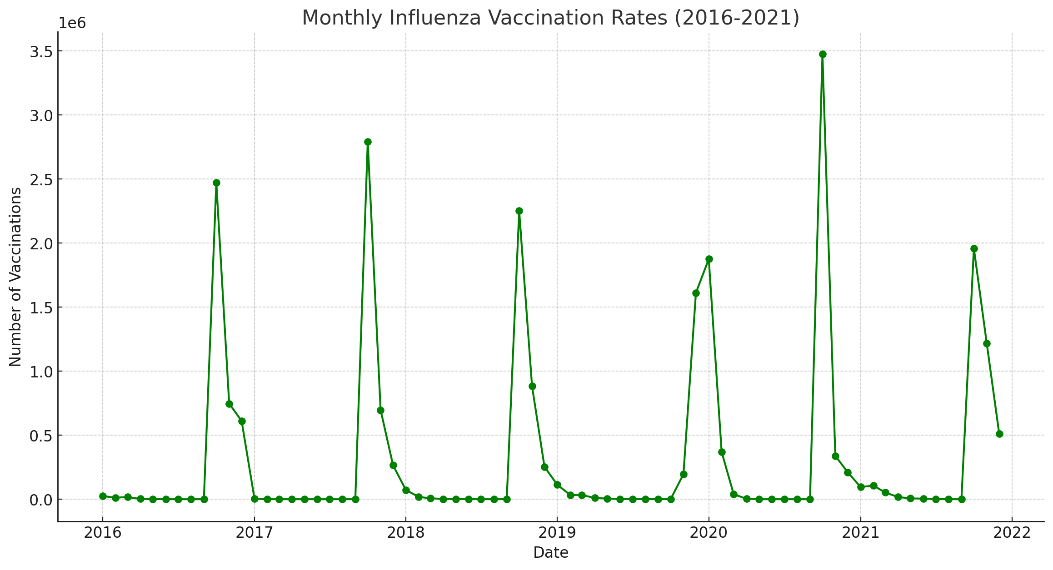 | 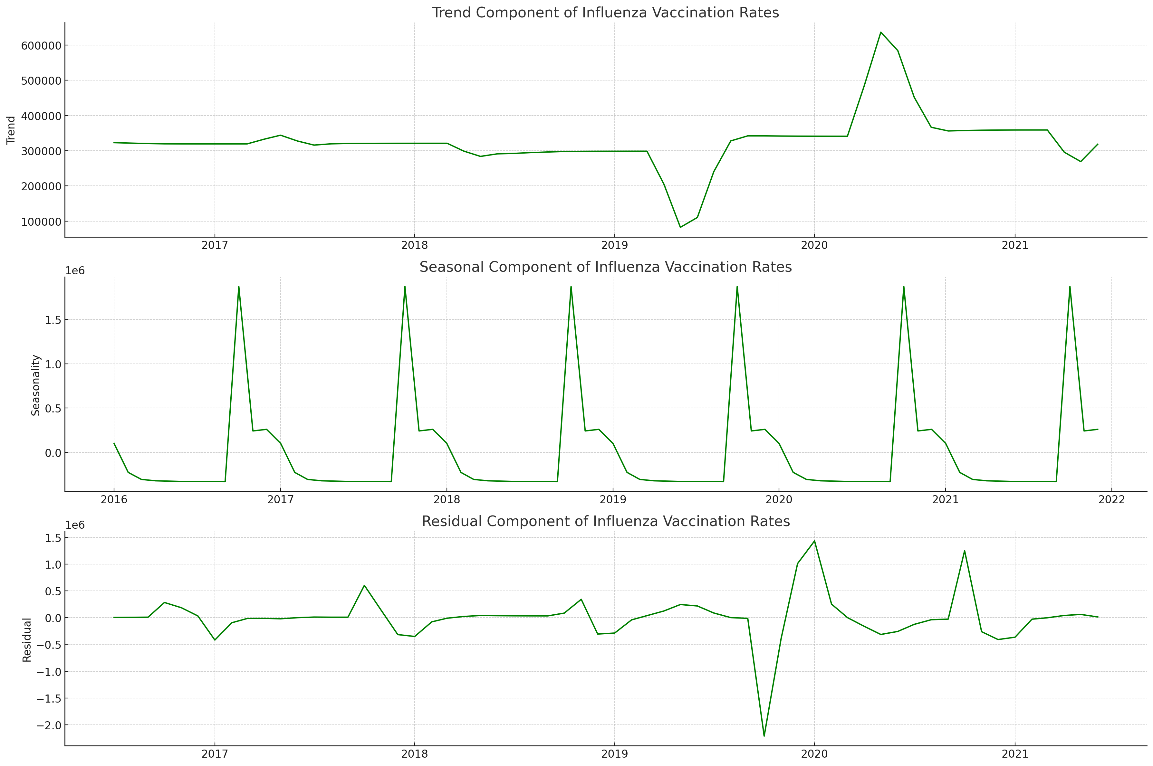 |
| --- | --- |
| Monthly rate of influenza vaccination from 2016 to 2021 (**Observed**) | Decomposition of the influenza vaccination data (**Decomposed**) |

The plot of monthly distribution of influenza vaccine administration shows the number of influenza vaccinations administered each month over the years. We can observe some peaks which suggest seasonal trends.

By conducting seasonal decomposition (right), the seasonal figure clearly highlights the annual seasonality, with peaks repeating around the same time each year, reflecting increased vaccine administration during typical flu seasons.

**Seasonality of rhabdomyolysis events**

| 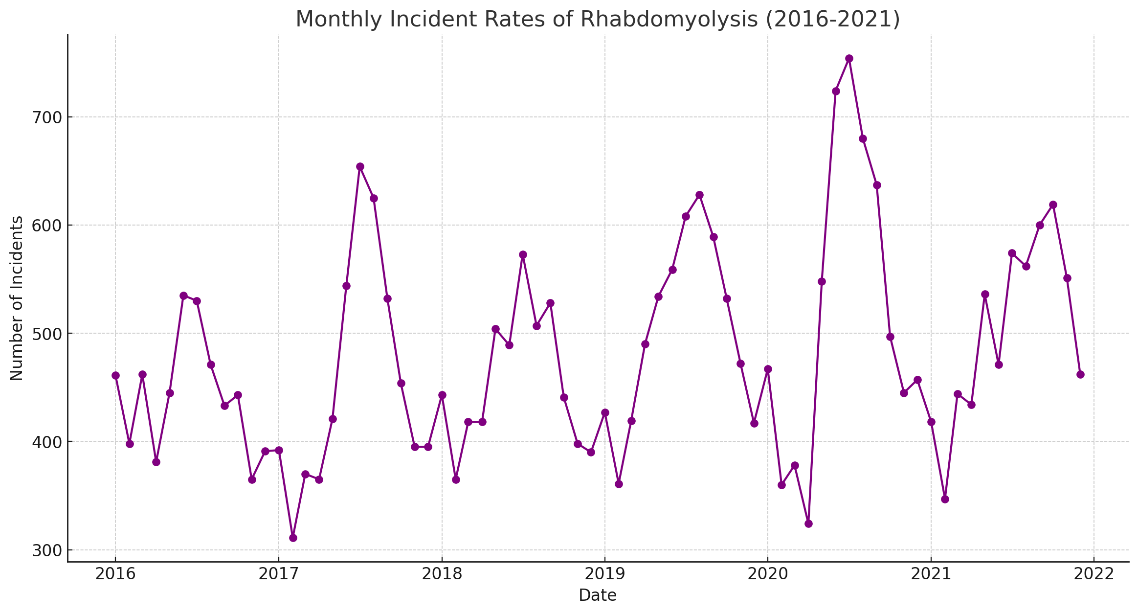 | 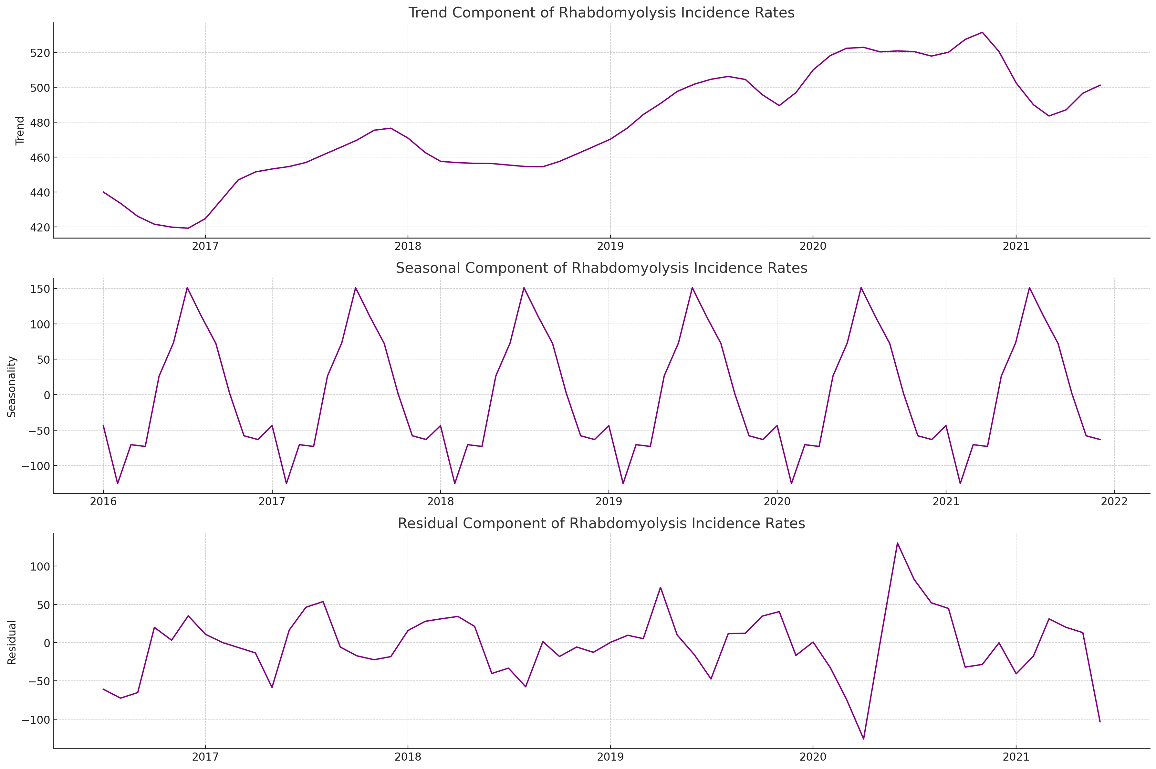 |
| --- | --- |
| Monthly rate of rhabdomyolysis from 2016 to 2021 (**Observed**) | Decomposition of the rhabdomyolysis data (**Decomposed**) |

The plot of monthly distribution of rhabdomyolysis events illustrates the monthly occurrences of rhabdomyolysis events. While there are some fluctuations, the seasonal pattern is less distinct than in the influenza vaccine data. The data shows variability throughout the years with some peaks that could suggest mild seasonal trends.

From the seasonal decomposition plots (right), the seasonal figure shows that seasonality is present but not as pronounced as in the influenza vaccine data. Peaks appear to be somewhat regular but less intense.

| ***Table*** ***S1.* Included statins.** | | |
| --- | --- | --- |
| **Drugs** | **ATC codes** | **Drug number** |
| Simvastatin | C10AA01 | A055967100, AB47348100, AC44998100, AC46402100, AC47775100, AC47907100, AC47924100, AC47928100, AC48608100, AC48813100, AC48926100, AC49190100, AC49360100, AC49535100, AC49661100, AC49672100, AC49699100, AC49792100, AC49841100, AC49997100, AC52465100, AC52479100, AC56804100, AC56806100, AC57176100, AC58207100, BC23970100, BC24339100, BC25211100 |
| Lovastatin | C10AA02 | A042389100, AC39307100, AC39403100, AC42539100, AC42558100, AC42627100, AC43573100 |
| Pravastatin | C10AA03 | A046022100, AB46029100, AB48586100, AB48644100, AB48681100, AB49021100, AB49143100, AB49454100, AB49503100, AC47341100, AC48469100, AC48513100, AC48684100, AC51523100, AC52581100, AC57126100, AC57741100, BC23596100, BC23597100 |
| Fluvastatin | C10AA04 | AC56629100, BC23556100, BC26147100 |
| Atorvastatin | C10AA05 | AA48879100, AA49226100, AA49288100, AA49543100, AA56739100, AA57774100, AA57950100, AB51732100, AB54967100, AB57772100, AB57967100, AB58049100, AC50086100, AC51598100, AC52301100, AC52530100, AC55268100, AC55272100, AC55583100, AC55895100, AC55952100, AC55956100, AC56319100, AC56682100, AC56791100, AC57133100, AC57267100, AC57805100, AC57930100, AC58041100, AC58211100, AC58366100, AC58401100, AC58517100, AC58579100, BA25200100, BA25201100, BA25337100, BC22886100, BC22889100, BC22890100, BC26028100, BC26350100 |
| Rosuvastatin | C10AA07 | AA57802100, AA57843100, AB57194100, AB57940100, AC57130100, AC57803100, AC57809100, AC57880100, AC58067100, AC58098100, AC58270100, AC58282100, AC58291100, AC58315100, AC58316100, AC58384100, AC58396100, AC58411100, AC58621100, AC58622100, AC58813100, AC58822100, AC58969100, AC59240100, AC59265100, AC59266100, AC59649100, AC59652100, AC60114100, AC60197100, BA25797100, BA25798100, BC24129100, BC24131100, BC24597100, BC26367100, BC26368100, BC26497100, BC26505100, BC26543100, BC26544100, BC26900100, BC27781100, BC27782100 |
| Pitavastatin | C10AA08 | AA57372100, AA58648100, AC58078100, AC58525100, AC58526100, AC58633100, AC58639100, AC59192100, AC59193100, AC59398100, AC60174100, AC60175100, AC60290100, AC60561100, BC25350100, BC27002100, BC28020100 |

| ***Table S2.* Risk factors for rhabdomyolysis.** | | | |
| --- | --- | --- | --- |
| **Risk factors** | **ICD-9 codes** | **ICD-10 codes** | **Time interval before index date** |
| Trauma | 800-999  (Except 840-848, 992.0-992.9, 994.3, 994.4, 994.5) | S00-S09 (Except S03.4, S03.8, S03.9, S09.11) S10-S19 (Except S13.4, S13.8, S13.9, S16.1) S20-S29 (Except S23.3, S23.4 S23.8, S23.9, S29.01)  S30-S39 (Except S33.5, S33.6 S33.8, S33.9, S39.01)  S40-S49 (Except S43.4, S43.5 S43.6, S43.8, S43.9, S46.01, S46.11, S46.21, S46.31, S46.81, S46.91,)  S50-S59 (Except S53.4)  S60-S69 (Except S63.5, S63.6 S63.8, S63.9), S70-S79 (Except S73.1, S76.01 S76.11, S76.21, S76.31, S76.81, S76.91)  S80-S89 (Except S83.4, S83.5 S83.6, S83.8, S83.9)  S90-S99 (Except S93.4, S93.5 S96.01, S96.11, S96.21, S96.81, S96.91)  T07, T14, T15-T19, T20-T25, T26-T28, T30-T32, T33-T34, T66-T78 (Except T67, T73), T79 | 7 days |
| Heat exposure | 992 | T67.0-T67.9 | 7 days |
| Hyperthermia | 333.92, 995.86 | G21.0, T88.3 | 7 days |
| Hypothyroidism | 243, 244 | E00, E02, E03, E06.3, E89.0 | 7 days |
| Myopathy | 359.0, 359.1, 359.2, 710.3, 729.1 | G71, M33, M60, M62, M63 | 7 days |
| Epilepsy | 345, 780.3 | G40, G41, R56 | 7 days |
| Stroke | 431, 432, 434, 435, 437, 362.34 | G45, G46, I61, I62, I63, I64, I67 | 90 days |
| Poisoning | 960-979, 980-989, 359.4 | T36-T50, T51-65, G72.0, G72.1, G72.2 | 90 days |
| Sepsis | 785.52, 995.92 | R65.21 | 14 days |
| Water deprivation | 276.5, 994.3, 994.4, 994.5 | E86, T73.1-T73.3 | 7 days |
| Electrolyte imbalances | 275.3, 276.8 | E83.3, E87.6 | 7 days |
| Fibrates* | C10AB01, C10AB02, C10AB04, C10AB05, C10AB09 | | 180 days |

*The codes for fibrates are ATC codes.

***Table S3*. Summary of case reports from literature regarding rhabdomyolysis following influenza vaccination**

| **Case no.** | **Sex / age** | **Onset of symptoms after influenza vaccination** | **Composition of the flu vaccine** | **Presented symptoms or signs** | **Concurrent or past use of statin** | **Other risk factors of rhabdomyolysis** | **Management and prognosis** |
| --- | --- | --- | --- | --- | --- | --- | --- |
| 1 | Male / 68 | 1 day | (Not reported) | Diffuse myalgia | Simvastatin 40 mg QD | Bezafibrate 200 mg QD | Forced diuresis and urine alkalization. Renal function returned to normal within the following days. |
| 2 | Male / 57 | 7 days | Inactivated split-virion vaccine (Aventis Pasteur) | Widespread muscle aches and difficulty | Simvastatin 20 mg QD for 6 years | (Not reported) | Increased dose of prednisolone. Serum creatine kinase value normalized on day 10 and muscle weakness gradually improved over several weeks. |
| 3 | Male / 70 | 1 day | Inactivated influenza virus (Imuvac) | Bilateral painful legs | Simvastatin 40 mg QD for 7 years | (Not reported) | Supportive treatment like  hemofiltration for several days. Serum creatine kinase level returned to within normal limits on day 6 and muscle weakness gradually improved over several months. |
| 4 | Male / 58 | 1 day | Influenza A H1N1 | Initially complained of mild myalgia. After four days, admitted with ascending weakness and intense myalgia in the lower back, as well as in the upper and lower limbs. | Simvastatin 40 mg QD | (Not reported) | Intravenous fluid therapy and hemodialysis several times. The patient was hemodynamically stable and asymptomatic  at hospital discharge. |

**References:**

1. Plotkin E, Bernheim J, Ben-Chetrit S, Mor A, Korzets Z. Influenza vaccine--a possible trigger of rhabdomyolysis induced acute renal failure due to the combined use of cerivastatin and bezafibrate. *Nephrol Dial Transplant.* 2000;15(5):740-741.

2. Raman KS, Chandrasekar T, Reeve RS, Roberts ME, Kalra PA. Influenza vaccine-induced rhabdomyolysis leading to acute renal transplant dysfunction. *Nephrol Dial Transplant.* 2006;21(2):530-531.

3. Shah SV, Reddy K. Rhabdomyolysis with acute renal failure triggered by the seasonal flu vaccination in a patient taking simvastatin. *BMJ Case Rep.* 2010;2010.

4. Callado RB, Carneiro TG, Parahyba CC, Lima Nde A, da Silva Junior GB, Daher Ede F. Rhabdomyolysis secondary to influenza A H1N1 vaccine resulting in acute kidney injury. *Travel Med Infect Dis.* 2013;11(2):130-133.

| ***Table S4.* Rhabdomyolysis risk associated with statin exposure within different risk windows, stratified by recent Influenza vaccination status; data from 2020-2021** | | | | | |
| --- | --- | --- | --- | --- | --- |
| **Influenza vaccine-exposed interval prior to the index date** | **Total number of cases** | **Number of cases**  **inside the statin-exposed interval** | **OR (95% CI)** | | **P-value** |
| **Statin risk window: 1-30 days prior to the index date** | | | | | |
| Individuals who received influenza vaccine prior to the index date | | | | | |
| 0–7 days prior | 35 | 14 | 1.37 (0.69 - 2.74) | | 0.371 |
| 8–14 days prior | 41 | 10 | 0.68 (0.33 - 1.41) | | 0.300 |
| Individuals who did NOT receive influenza vaccine prior to the index date | | | | | |
| 0–7 days prior | 2,203 | 788 | 1.07 (0.98 - 1.17) | 0.135 | |
| 8–14 days prior | 2,197 | 792 | 1.08 (0.99 - 1.18) | 0.080 | |
| **Statin risk window: 1-60 days prior to the index date** | | | | | |
| Individuals who received influenza vaccine prior to the index date | | | | | |
| 0–7 days prior | 35 | 14 | 1.32 (0.66 - 2.63) | | 0.437 |
| 8–14 days prior | 41 | 11 | 0.75 (0.37 - 1.50) | | 0.411 |
| Individuals who did NOT receive influenza vaccine prior to the index date | | | | | |
| 0–7 days prior | 2,203 | 809 | 1.07 (0.98 - 1.17) | 0.149 | |
| 8–14 days prior | 2,197 | 812 | 1.08 (0.99 - 1.18) | 0.098 | |
